# Disseminated Invasive Aspergillosis in a Young Patient With Chronic Alcohol Use and Seemingly Preserved Immunocompetence: A Case Report

**DOI:** 10.1002/ccr3.71943

**Published:** 2026-01-24

**Authors:** Kumail Khandwala, Hiba Sawliha Syed, Shayan Sirat Maheen Anwar, Sehar Suleman, Khabab Abbasher Hussien Mohamed Ahmed

**Affiliations:** ^1^ Department of Radiology Aga Khan University Karachi Pakistan; ^2^ Department of Pathology Aga Khan University Karachi Pakistan; ^3^ Faculty of Medicine University of Khartoum Khartoum Sudan

**Keywords:** alcoholism, fungal pneumonia, immunocompetent host, invasive aspergillosis, malignancy

## Abstract

Invasive aspergillosis, though typically seen in immunocompromised patients, can also affect immunocompetent individuals and mimic pulmonary malignancy. This case highlights the importance of considering fungal infections in the differential diagnosis of chronic respiratory symptoms, particularly in patients with risk factors such as heavy alcohol consumption, that may impair immune function.

## 1 Introduction

Invasive aspergillosis (IA) is most frequently described in profoundly immunocompromised hosts, including those with hematologic malignancies, neutropenia, solid organ or hematopoietic stem cell transplantation, and advanced HIV infection. However, emerging evidence demonstrates that IA may occur in immunocompetent or immune‐impaired individuals [1, 2, 3, 4, 5, 6].

The global incidence and disease burden of severe fungal disease have risen and are now recognized as a major public health threat [2, 7].

Disseminated aspergillosis can be defined as infection involving at least two noncontiguous organ systems; it is uncommon, but as one study demonstrates, it is associated with an all‐cause mortality of up to 30% [8]. The lungs are generally the primary portal of entry, with hematogenous spread to the CNS, cardiovascular system, kidneys, and liver. Cardiac aspergillosis most often manifests as endocarditis or pericarditis, while CNS involvement includes meningitis, encephalitis, or intracranial abscess. Pulmonary aspergillosis is the most common presentation, ranging from focal consolidation to mass‐like lesions, cavitation, and airway‐invasive patterns that can mimic tuberculosis or malignancy [9, 10]. Hepatic aspergillosis, though rare, can present as liver abscesses or multifocal hepatic lesions, usually in the context of disseminated disease [11].

Alcohol misuse is a significant, yet frequently overlooked, risk factor for invasive aspergillosis (IA). Research indicates that chronic alcohol consumption causes comprehensive respiratory and immune dysfunction, specifically impairing mucociliary clearance and predisposing patients of aspiration [12]. Furthermore, ethanol exposure induced transcriptional and epigenetic changes that drive monocyte and macrophage dysfunction, simultaneously dampening essential T‐cell responses [13, 14]. These combined effects create an environment, though subtle compared to classic immunosuppression, that is conducive to fungal colonization and invasion [7].

We present a rare case of invasive aspergillosis in a young man with no known classical immunosuppressive conditions but a history of heavy alcohol use. This case highlights the diagnostic challenge of fungal infections mimicking malignancy and the need to broaden differential diagnoses in patients with chronic respiratory symptoms.

## 2 Case Presentation

### 2.1 History and Clinic Presentation

A male in his early 30s with no known comorbidities and a history of heavy episodic alcohol consumption presented with a year‐long cough, fever, weight loss, and headaches to pulmonology and infectious disease clinic. By occupation, our patient worked as a receptionist at a clinical laboratory, occasionally handling patient samples for transportation. He had no clear history of inoculation injury. Imaging performed prior to presentation showed a right lung mass. Considering the chronicity of his symptoms and no identifiable risk factors, the primary differential for this lung mass was malignancy, at the time suspected to be a sarcoma. He was referred for a computed tomography‐guided targeted lung biopsy.

Subsequently, histopathology revealed lung tissue core showing fungal infection with giant cell reaction, focal granulomatous inflammation, and stromal collagenization. The patient presented to the pulmonology clinic on follow‐up and, due to acutely exacerbated pulmonary distress, was immediately referred to the emergency department.

### 2.2 Emergency Department Presentation

**FIGURE 1:**
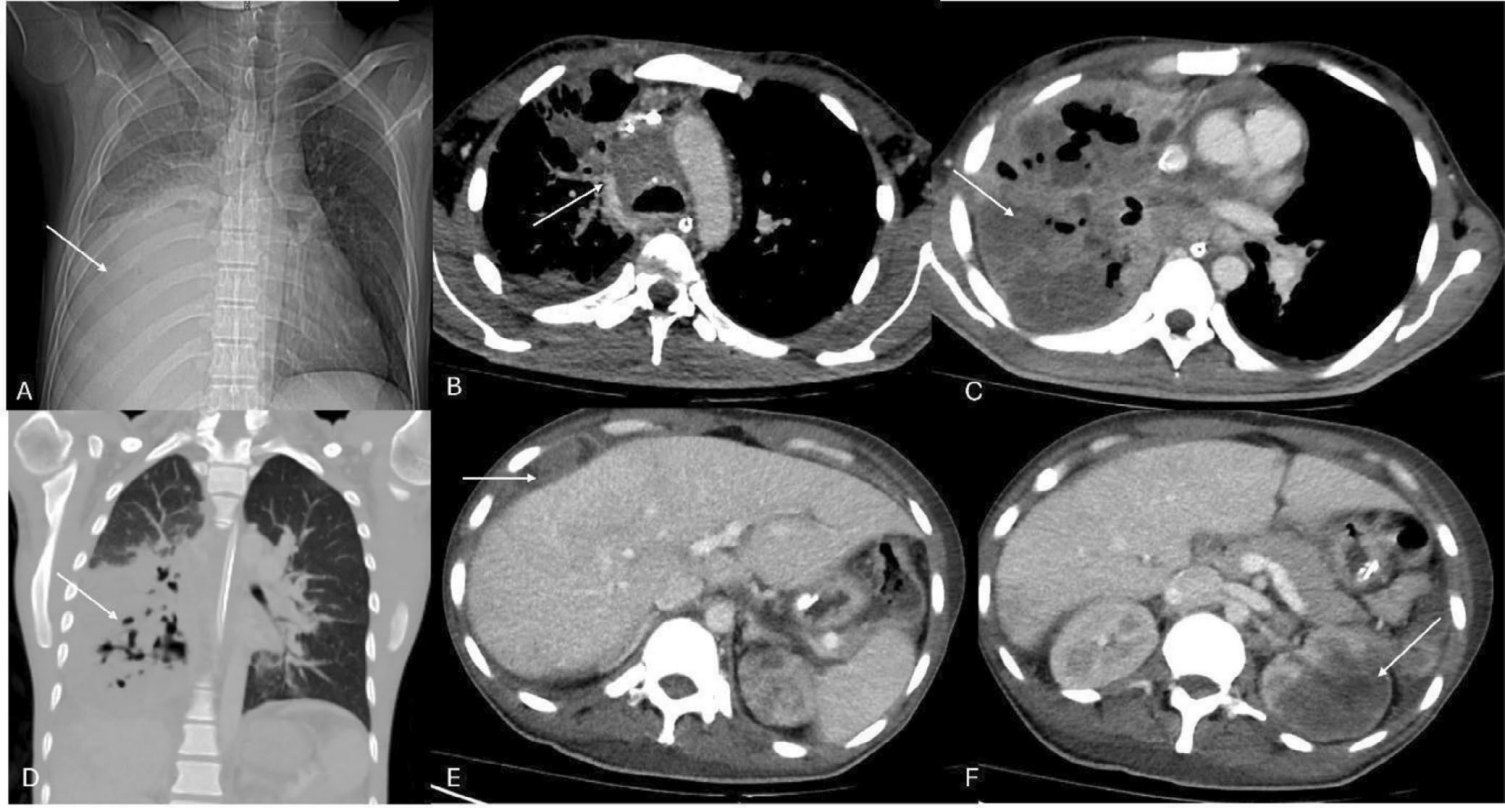
(A) Chest x‐ray showing large consolidative opacity in the right lower lung zone (arrow). (B, C) Axial CT chest image showing multifocal low attenuation infiltrative masses in the mediastinum and right lung (arrows). (D) Areas of cavitation and necrosis demonstrated on coronal CT image in lung window settings (arrow). (E, F) Axial CT images from the upper abdomen showed perihepatic lesions (arrow in E) and left renal lesions (arrow in F).

He presented to the emergency department with fever, cough, and shortness of breath. An urgent chest x‐ray was advised due to progressively worsening dyspnea which showed homogenous soft tissue opacification of the right middle and lower lung zones, silhouetting the right heart border and hemi‐diaphragm with some air bronchograms, and an enlarged heart shadow was also noted (Figure 1).

His baseline workup showed severe metabolic acidosis, severe hyponatremia, acute kidney injury, severe anemia, leukocytosis with lymphopenia and neutrophilia, marked thrombocytosis (leucoerythroblastic peripheral film), prolonged coagulation parameters, and markedly elevated D‐dimer levels. Importantly, the coagulation profile did not fulfill criteria for classical consumptive disseminated intravascular coagulation, which is typically characterized by thrombocytopenia and hypofibrinogenemia. Instead, the constellation of findings was consistent with disseminated intravascular activation and a systemic inflammatory response, likely driven by severe infection and marrow stress. These laboratory findings together raised suspicion for multi‐organ dysfunction and disseminated disease (Table 1).

**TABLE 1:** Laboratory workup at presentation in the ER.

Tests	Results	Normal ranges/notes
Arterial blood gas		
pH	7.26	7.35–7.45
PCO_2_	15.2 mmHg	35–48
PO_2_	58.1 mmHg	83–108
Bicarbonate	5.7 mmol/L	22–28
Lactate	> 14 mmol/L	Venous: 0.56–1.39 mmol/L (at bed rest) Arterial: 0.36–0.75 mmol/L (at bed rest)
Serum glucose/electrolytes and renal function tests		
Sodium	125 mmol/L	136–145
Potassium	5.7 mmol/L	3.5–5.1
Magnesium	3.0 mg/dL	1.6–2.6
Ionized calcium	4.68 mg/dL	4.64–5.28
Chloride	93 mmol/L	98–107
Blood urea Nitrogen (BUN)	26 mg/dL	6–20
Creatinine	2.5 mg/dL	0.9–1.3
Serum glucose (random)	140 mg/dL	80–160
Complete blood count		
Hemoglobin	3.9 g/dL	12.3–16.6
Hematocrit	14.2%	38.4–50.7
White cell count	26.1 × 10E9/L	4.8–11.3
Lymphocytes	10%	17.5–45
Neutrophils	75%	34.9–76.2
Platelets	593 × 10E9/L	154–433
Peripheral film		Dimorphic red cells with anisocytosis, hypochromia, microcytosis, poikilocytosis, polychromasia, and nucleated RBCs. Platelets increased. Myelocytes and metamyelocytes present (leucoerythroblastic picture) with monocytosis and neutrophilia. Suggests marrow stress; further workup advised.
Infective markers		
Procalcitonin	1.43 ng/mL	Procalcitonin levels < 0.5 ng/mL = low risk of severe sepsis and/or septic shock. Procalcitonin levels > 2.0 ng/mL = high risk of severe sepsis and/or septic shock.
Coagulation profile		
Prothrombin time	17.6 s	9.3–12.8
International normalized ratio (INR)	1.7	0.9–1.2
Activated partial thromboplastin time (aPTT)	65.5 s	22.9–34.5
D‐dimer	23.5 mg/L FEU	< 0.5
Fibrin‐degradation product	> 20 μg/mL	< 5
Fibrinogen level	543 mg/dL	156–400
Cardiac enzymes		
Troponin‐I	10 ng/dL	0–57 (males)
Liver function tests		
Total bilirubin	0.9 mg/dL	0.1–1.2
Direct bilirubin	0.6 mg/dL	0–0.2
Indirect bilirubin	0.3 mg/dL	0.1–0.8
GGT	112 IU/L	Male: < 55
SGPT (ALT)	131 IU/L	Male: < 45
SGOT (AST)	813 IU/L	Male: < 35
AP	587	45–129
Serum albumin	2.8 g/dL	3.5–5.2

### 2.3 Differential Diagnosis

In the outpatient clinic, the chronicity of cough, fever, and weight loss together with the mass‐like lesion on imaging raised suspicion for pulmonary malignancy such as sarcoma, and in a high tuberculosis‐burden setting, pulmonary tuberculosis was also strongly considered. Hematologic malignancy was initially a primary consideration given the patient's leucoerythroblastic blood film. However, because IA in non‐neutropenic hosts frequently presents as space‐occupying lesions that mimic primary lung cancer, other fungal pathogens such as mucormycosis and histoplasmosis were maintained in the differential. Recent literature emphasizes that IA should be considered early in the diagnostic workup of mass‐like pulmonary lesions, even in patients who lack traditional iatrogenic immune suppression [5, 6, 9, 10, 15]. However, by the time the patient presented to the emergency department, biopsy had already demonstrated fungal elements, and subsequent serum fungal markers were positive, narrowing the working diagnosis to invasive fungal infection. At that point, the differential shifted from questioning whether the process was malignant or infectious to characterizing the extent of dissemination and excluding other fungal etiologies and evaluating underlying risk factors with culture subsequently confirming *Aspergillus flavus* as the causative organism.

### 2.4 Assessment of Host Immune Status and Fungal Biomarkers

An investigation into possible underlying immunocompromising disease was launched, including human immunodeficiency virus (HIV) assay, hepatitis B surface antigen and hepatitis C virus antibody, which were all negative.

An abdominal ultrasound demonstrated a normal‐sized liver with regular margins and no stigmata of chronic liver disease.

On lymphocyte subset analysis, absolute counts of CD3+ T‐lymphocytes and CD19+ B‐lymphocytes were normal. Both CD4+ helper and CD8+ cytotoxic T‐cell subsets were within normal limits with a preserved CD4+/CD8+ ratio. CD56+ natural killer (NK) cells were also normal.

These findings indicate an intact adaptive and innate immune profile with no evidence of cellular immunodeficiency. This supports classification of the patient as immunocompetent by conventional laboratory criteria, despite functional impairment from chronic alcohol use.

Serum Beta D glucan (BDG) and Galactomannan index (GALA) were strongly positive, as was expected.

Immunoglobulins were tested for hematologic pathology but were not indicative of such. There was no evidence of hypogammaglobulinemia or immunodeficiency. Elevated IgA and IgG suggested a reactive response to ongoing systemic infection (disseminated aspergillosis) rather than an underlying immunosuppressive state (Table 2).

**TABLE 2:** Immune status testing.

Test	Result	Normal range/notes
Viral serology/infectious disease screening		
HIV assay	Negative	
HIV enzyme immunoassay (EIA)	Non‐reactive	
Hepatitis B surface antigen	Non‐reactive	
Hepatitis C virus antibody assay	Non‐reactive	
Lymphocyte subset analysis		
Absolute count of CD3+ total T‐lymphocytes	959.0	725–3171
Absolute count of CD4+ T‐helper lymphocytes	519.0	492–2014
Absolute count of CD8+ T‐regulatory lymphocytes	424.0	200–1541
Absolute count of CD19+ Total B lymphocytes	183.0	Normal range 104–1035
Absolute count of CD56+ natural killer cells	246.0	56–724
Immunoglobulins		
Serum IgA	7.01 g/L	0.7–4
Serum IgM	0.72 g/L	0.4–2.3
Serum IgG	22.2 g/L	7–16

### 2.5 Imaging Evaluation and Extent of Dissemination

Extensive cross‐sectional and echocardiographic imaging confirmed disseminated multi‐organ involvement.

A transthoracic echocardiogram revealed mildly reduced left ventricular systolic function (ejection fraction ≈45%) with a moderate to large circumferential pericardial effusion. An echogenic mobile focus was visualized on the mitral valve chordal apparatus, consistent with a vegetation, highly suspicious for fungal endocarditis [16] in the clinical context.

Contrast‐enhanced CT of the chest demonstrated collapse consolidation of the right lung with sparing of the upper lobe. Multiple lobulated peripherally enhancing lesions were identified in the right middle and lower lobes, several with central necrosis, air pockets, and air bronchograms [9, 10]. These extended into the posterior mediastinum and prevertebral space, encasing the right main pulmonary artery. There was diffuse right parietal pleural enhancement and associated patchy consolidation in the contralateral lung. Septal thickening and ground‐glass opacities were noted in the right upper lobe.

In the abdomen and pelvis, CT revealed multifocal visceral involvement. A peripherally enhancing hypodense lesion was present in the right perihepatic region with subtle hypodensities in the right hepatic lobe. A large heterogeneous lesion involving the interpolar and lower pole of the left kidney with extension into the pelvicalyceal system was identified, along with an additional small lesion near the renal hilum. Mild to moderate ascites with peritoneal thickening was also present. These findings were consistent with disseminated invasive aspergillosis with hepatic and renal abscess formation, a recognized manifestation of hematogenous fungal spread (Figure 1).

**FIGURE 2:**
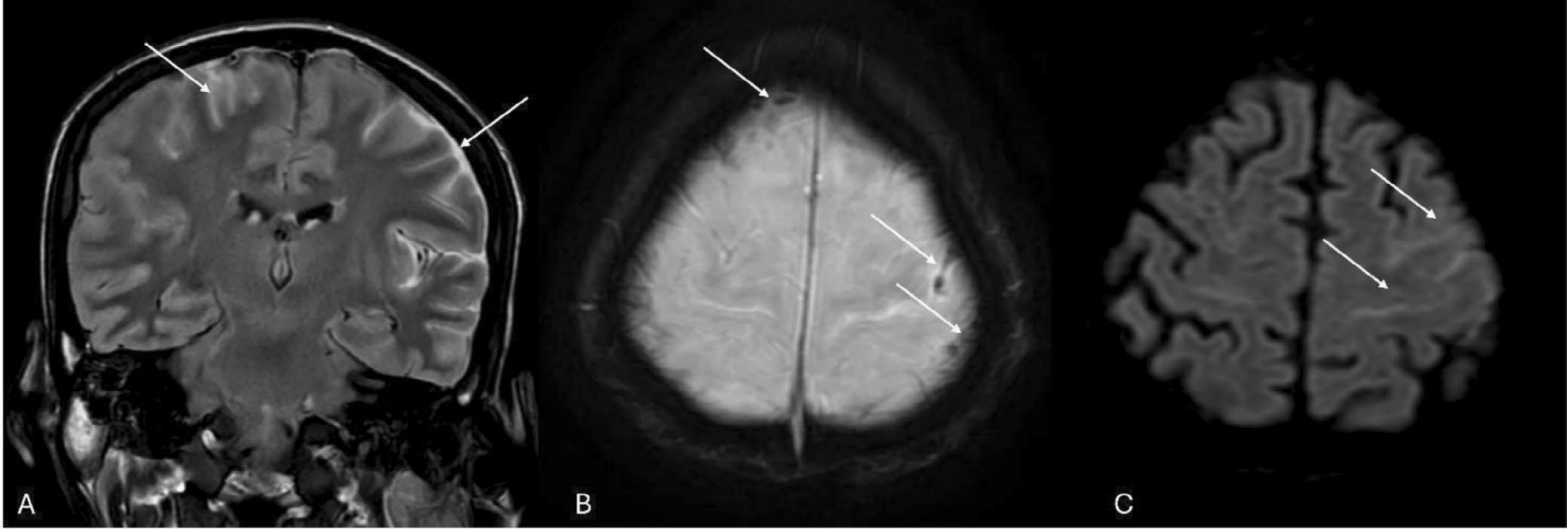
MR images of the brain. (A) FLAIR coronal post‐contrast image showing diffuse sulcal hyperintensity, more on the left side (arrows). (B) Susceptibility weighted image shows areas of dropout in the sulcal spaces (arrows), likely attributable to paramagnetic properties of fungal elements and arachnoiditis. (C) Subtle corresponding diffusivity also noted on diffusion‐weighted axial image (arrow).

Magnetic resonance imaging of the brain showed diffuse sulcal FLAIR hyperintensity most marked over the left cerebral convexity with scattered areas of susceptibility on SWI, positive diffusion restriction, and post‐contrast meningeal enhancement. These appearances suggested meningoencephalitis with arachnoiditis [9], although a component of subarachnoid hemorrhage with reactive meningeal enhancement could not be entirely excluded. No focal parenchymal abscess or space‐occupying lesion was identified (Figure 2).

Lumbar puncture was not performed due to coagulopathy and critical illness.

Additionally, venous Doppler of the left upper limb demonstrated a partially occluding thrombus in the brachial vein, reflecting the prothrombotic state of systemic invasive fungal infection.

Together, these multimodality findings confirmed widespread dissemination of *Aspergillus flavus* infection with pulmonary, mediastinal, pleural, renal, hepatic, central nervous system, and cardiac involvement.

### 2.6 Management and Treatment

A multidisciplinary approach to care was established; infectious disease, internal medicine, nephrology, neurology, and pulmonology were involved, and he was managed with antifungal optimization, intubation–ventilation, and hemodialysis; subsequently, he was admitted to the intensive care unit. Thorough imaging was performed to ascertain the extent of the disease.

Based on biopsy findings, eventually strongly positive BDG/Galactomannan, and disseminated disease, antifungal monotherapy was initiated with oral voriconazole (200 mg twice daily) which was considered reasonable in this setting as it has good CNS penetration [17] and is the Infectious Diseases Society of America (IDSA) recommended first‐line agent for invasive aspergillosis, with combination therapy reserved for refractory or rapidly progressive disease [18, 19]. Amphotericin B was avoided due to contraindication in the setting of acute kidney injury.

### 2.7 Conclusion and Results

**FIGURE 3:**
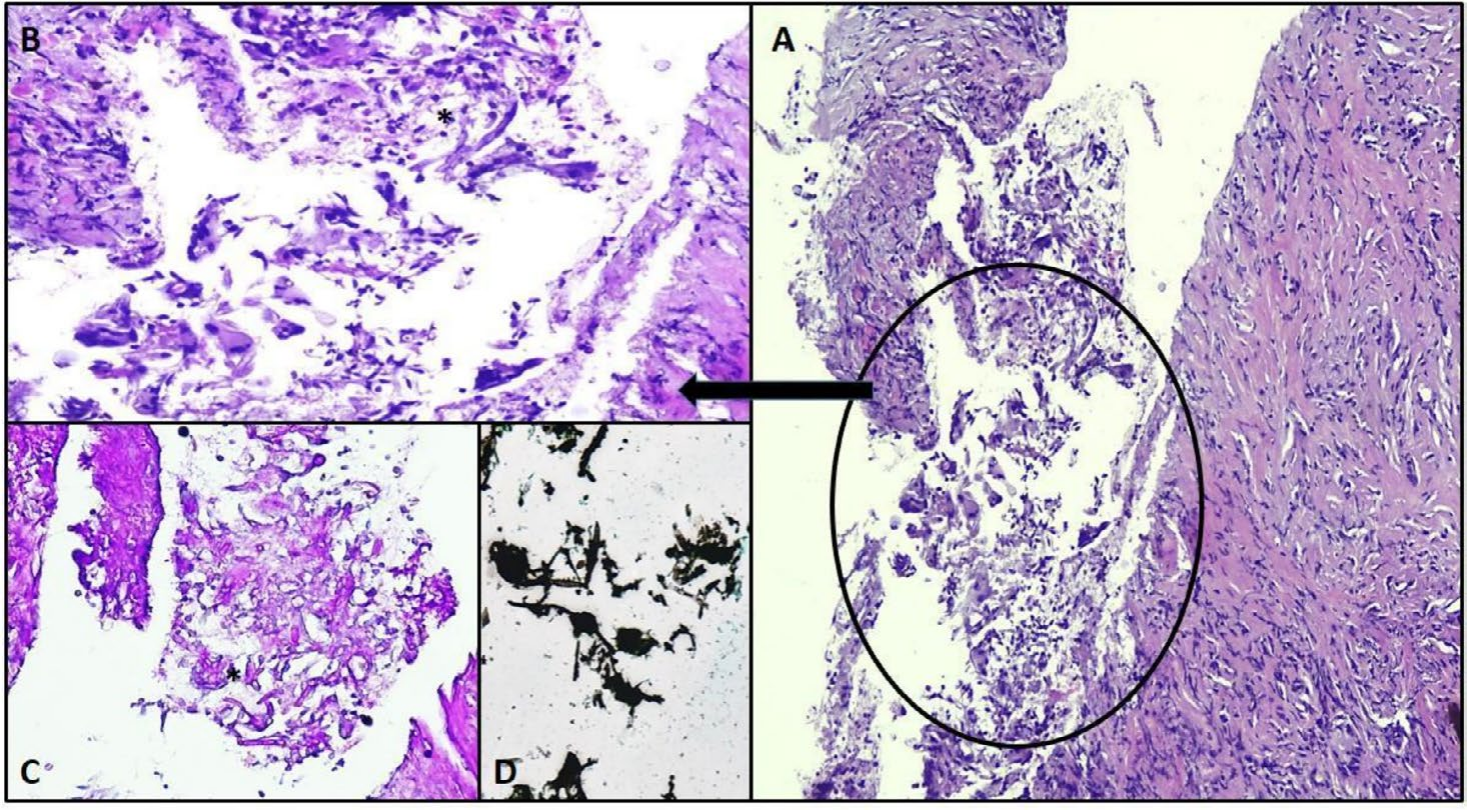
Histology slides of tissue from lung: Photomicrographs (A) and (B) show low and high magnification H&E stained slides, at 10 and 20×, respectively, of lung parenchymal tissue exhibiting mixed inflammatory cells along with multinucleated giant cells elicited by aggregates of non‐branching septate fungal hyphae (A, B). Two special stains periodic acid–Schiff (PAS) in photomicrographs (C) highlight these fungal hyphae (C), while Gomori's methenamine silver (GMS) stain in (D) highlights both fungal hyphae and associated tissue reaction.

On histopathology (Figure 3) fungal spores and hyphae with giant cell reaction, focal granulomatous inflammation and stromal collagenization were described, and no evidence of malignancy was seen. The following day tracheal culture reported growth of *Aspergillus flavus*. His response to voriconazole was monitored by a steadily declining BDG and GALA. Despite the patient's hospital course being complicated by ventilator‐associated pneumonia, he was ultimately extubated; care was progressively downgraded, and he was discharged to be managed as outpatient. Serial chest x‐rays demonstrate progressive reduction in pulmonary consolidation.

He was discharged on a prolonged oral voriconazole course. Unfortunately, he was lost to follow‐up after discharge.

## 3 Discussion

Invasive aspergillosis (IA) is classically linked to profound immunosuppression, yet contemporary cohorts show a substantial burden among non‐neutropenic or otherwise “immunocompetent‐by‐labs” patients. In a modern tertiary care cohort, a sizable fraction of IA occurred without neutropenia, underscoring that neutropenia is no longer the sole or dominant risk in many settings. This shift has implications for clinical suspicion and earlier use of fungal biomarkers and imaging in atypical hosts [4, 17].

Our patient exemplifies the evolving landscape of IA, where neutropenia is no longer the sole dominant risk factor [4, 20, 21]. Despite having normal lymphocyte subsets and neutrophil counts, his history of binge‐pattern alcohol use likely resulted in functional immune impairment. Current evidence suggests that alcohol disrupts the gut–lung axis and barrier integrity, allowing for increased systemic vulnerability [22]. These functional derangements characterized by impaired innate defenses and altered immune signaling lowered the threshold for angioinvasive disease and subsequent hematogenous spread to the CNS, heart, and kidneys, even in the absence of “on‐paper” immunosuppression [4, 14, 17].

Dissemination biology helps clarify the multi‐organ pattern we observed. After inhalational entry and pulmonary invasion, aspergillus can breach epithelial and endothelial barriers, enter the bloodstream (aided by hyphal angioinvasion), and seed distant organs [20]. Host phagocytes attempt clearance within the vasculature; however, when fungal burden or virulence factors overwhelm these defenses, organisms cross endothelium and establish foci in the CNS, heart, kidneys, and liver, precisely the distribution seen here.

CNS involvement in IA ranges from meningitis and encephalitis to hemorrhagic or abscess‐forming lesions. Our MRI showed sulcal FLAIR hyperintensity with SWI susceptibility and meningeal enhancement, compatible with meningoencephalitis/arachnoiditis without a focal abscess. Comparable CNS disease in immunocompetent or minimally immunosuppressed hosts has been described and carries high morbidity [9, 21], reinforcing the need for early neuroimaging when headache or altered mentation co‐exist with pulmonary IA.

Cardiac involvement is uncommon but devastating [15]. The patient's TEE revealed a mobile echogenic mass adherent to the mitral chordae plus a sizable pericardial effusion. These findings fit aspergillus endocarditis/pericarditis in the right clinical context. Recent reviews emphasize the diagnostic difficulty (often culture‐negative blood), frequent embolic complications, and the benefit of combining echocardiography with fungal biomarkers and tissue diagnosis whenever feasible [16].

Beyond our single case, the literature documents recurrent or disseminated IA in immunocompetent individuals, including reports of repeated invasive disease and multisystem lesions without classic risk factors. These cases echo our message: functional or situational immune impairments (e.g., alcohol‐related, critical illness, viral pneumonias) can suffice for angioinvasion and spread [5, 6, 7].

Therapeutically, voriconazole [17, 18] remains first line for proven/probable IA due to superior outcomes versus amphotericin in pivotal trials and guideline recommendations; combination regimens are reserved for select severe or refractory cases or when resistant species are suspected. In our patient, oral voriconazole (200 mg BID) was chosen; amphotericin B was avoided because of acute kidney injury, and the patient clinically improved with declining BDG/Galactomannan before being discharged (later lost to follow up).

Finally, a pragmatic diagnostic lesson: IA frequently mimics malignancy on imaging. In high TB‐burden regions, the differential is often narrowed to TB versus cancer, delaying fungal work‐up. Early consideration of IA, strategic use of serum BDG/Galactomannan, and tissue sampling can prevent delays, especially when lesions cavitate, cross tissue planes, or track along the mediastinum and pleura as seen here.

IA can occur, and disseminate, in patients who test “immunocompetent” on paper. Functional risk factors like chronic alcohol use, the biology of angioinvasion, and subtle or protean imaging findings demand a high index of suspicion, comprehensive staging for CNS and cardiac disease, and timely initiation of triazole therapy, tailored to renal/hepatic constraints.

## 4 Conclusion

- The risk factors and predisposing conditions for invasive fungal disease include but are not limited to classical immunocompromised states.
- The imaging findings of invasive aspergillosis are varied and often mimic malignancies.
- With the rising incidence of fungal infections, early diagnosis with imaging and serum fungal markers and subsequent timely management are instrumental in producing positive outcomes.

## Author Contributions

**Kumail Khandwala:** conceptualization, data curation, formal analysis, funding acquisition, investigation, methodology, project administration, resources, software, supervision, validation, visualization, writing – original draft, writing – review and editing. **Hiba Sawliha Syed:** investigation, resources, validation, visualization, writing – original draft, writing – review and editing. **Shayan Sirat Maheen Anwar:** conceptualization, investigation, methodology, project administration, validation, visualization, writing – original draft, writing – review and editing. **Sehar Suleman:** conceptualization, investigation, validation, visualization, writing – original draft, writing – review and editing. **Khabab Abbasher Hussien Mohamed Ahmed:** conceptualization, supervision, validation, visualization, writing – original draft, writing – review and editing.

## Funding

The authors have nothing to report.

## Ethics Statement

Ethical approval was obtained from the Ethics Review Committee, Aga Khan University, Pakistan (2024‐10613‐31003).

## Consent

Written informed consent was obtained from the patient to publish this report in accordance with the journal's patient consent policy.

## Conflicts of Interest

The authors declare no conflicts of interest.

## Data Availability

All the data that support the findings of this study are available from the corresponding author upon reasonable request.
